# Caught Between the Brain and a Hard Place: A Case of a Cervicomedullary AVM

**DOI:** 10.1155/crra/2571688

**Published:** 2026-04-16

**Authors:** A. Rumhumha, R. Minty, A. Lupuwana, A. Ranchod, W. Nkosi, M. Ramantsi

**Affiliations:** ^1^ Department of Diagnostic Radiology, University of the Witwatersrand, Johannesburg, South Africa, wits.ac.za; ^2^ Department of Diagnostic Radiology, NRS Incorporated, Netcare N17 Private Hospital, University of the Witwatersrand, Johannesburg, South Africa, wits.ac.za; ^3^ Department of Diagnostic Radiology, Chris Hani Baragwanth Academic Hospital, University of the Witwatersrand, Johannesburg, South Africa, wits.ac.za

**Keywords:** arteriovenous malformation, cervicomedullary junction, digital subtraction angiography, Gamma Knife, juvenile AVM, posterior fossa

## Abstract

Posterior fossa arteriovenous malformations (AVMs) are uncommon lesions that carry significant risk due to their proximity to the brainstem. We present the case of an 18‐year‐old female who suffered a sudden severe headache and was found to have a subarachnoid haemorrhage caused by a cervicomedullary junction AVM of the juvenile Type IIa subtype. Multimodality imaging with noncontrast CT, MRI and digital subtraction angiography (DSA) confirmed a posterior fossa AVM centred at the cervicomedullary junction. The patient was managed conservatively with strict blood pressure control and analgesia; Gamma Knife radiosurgery was considered but deferred given the diffuse nidus and the lesion′s location adjacent to the medulla. This case illustrates the diagnostic and therapeutic challenges of posterior fossa AVMs in young patients and highlights the crucial role of multimodality imaging in guiding management decisions.

**Learning Points**

• Posterior fossa arteriovenous malformations (AVMs) are rare lesions but carry a high risk of rupture, especially when located at the cervicomedullary junction.
• Juvenile Type IIa AVMs (Lasjaunias classification) are large, diffuse and fed by multiple arteries, making treatment particularly challenging.
• Multimodality imaging is essential: Noncontrast CT detects acute haemorrhage, MRI defines the nidus and its relationship to critical structures, and digital subtraction angiography (DSA) provides detailed vascular anatomy as the gold standard.
• Management requires careful, individualised planning—balancing the risks of intervention (surgery, embolisation or radiosurgery) against the risks of the lesion′s natural history.
• Early recognition of AVM rupture and timely referral to specialised centres are vital, especially in young patients, to optimise outcomes.
• High‐grade AVMs in eloquent locations may be best managed conservatively with structured longitudinal surveillance.

## 1. Introduction

AVMs are congenital tangles of arteries and veins that connect directly through a nidus, bypassing the capillary network. This high‐flow shunt can stress nearby vessels and brain tissue, increasing the likelihood of rupture. Posterior fossa AVMs are relatively rare but have a higher risk of bleeding and worse outcomes than supratentorial AVMs because of their location near the brainstem and cranial nerves [1, 2]. Juvenile AVMs, as defined in the Lasjaunias classification, form a distinct subgroup of particularly complex lesions. In this classification, Type IIa ‘juvenile’ AVMs are usually large, have multiple arterial feeders and exhibit diffuse venous outflow [3]. These features make them very difficult to treat effectively. We present an unusual case of a juvenile Type IIa AVM at the cervicomedullary junction in an 18‐year‐old woman, highlighting the value of multimodality imaging and the challenging balance between intervention and conservative management.

## 2. Case Report

An 18‐year‐old female, previously healthy, presented to the emergency department with a sudden, severe occipital headache. She described it as the worst headache of her life, accompanied by nausea, photophobia and neck stiffness. On examination, she was alert and orientated, with signs of meningism but no focal neurological deficit.

**Figure 1:**
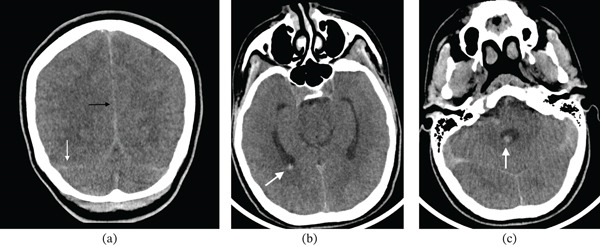
Noncontrast CT of the brain (axial images). (a) Acute subarachnoid haemorrhage along the falx cerebri (black arrow) and tentorium cerebelli (white arrow). (b) Acute intraventricular haemorrhage in the atrium of the right lateral ventricle (white arrow). (c) Acute intraventricular haemorrhage in the fourth ventricle (white arrow).

Lumbar puncture yielded cerebrospinal fluid (CSF) with elevated protein and more than 10,000 red blood cells/mm^3^, consistent with subarachnoid haemorrhage. A noncontrast CT of the brain demonstrated acute subarachnoid haemorrhage along the falx cerebri, the tentorium cerebelli and the premedullary cistern. There was also acute intraventricular haemorrhage in the atrium of the right lateral ventricle and in the fourth ventricle, with sulcal effacement indicating raised intracranial pressure (Figure 1a, b. and c).

**Figure 2:**
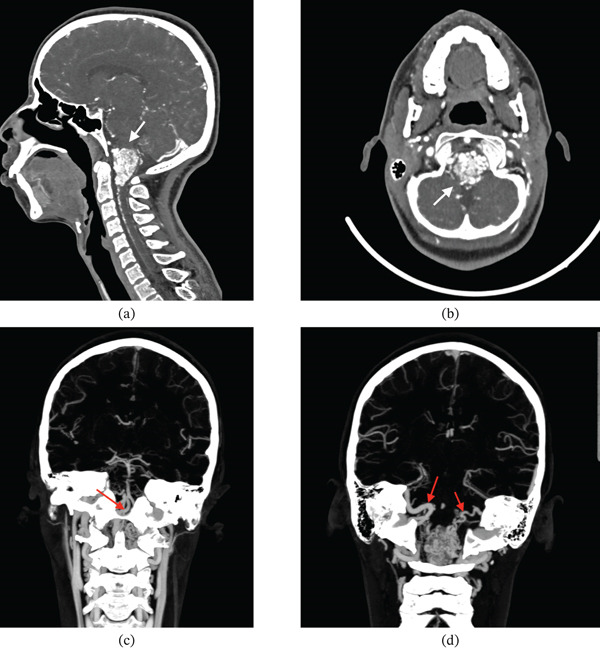
CT angiography demonstrating an arteriovenous malformation nidus at the cervicomedullary junction with serpiginous enhancing vessels. (a) Sagittal reformatted image showing the nidus (white arrow). (b) Axial image confirming the nidus at the cervicomedullary junction (white arrow). (c) Arterial supply arising from the anterior spinal artery (red arrow). (d) Early venous drainage into the bilateral sigmoid sinuses (red arrows).

Subsequently, a CT angiogram (CTA) of the brain and neck revealed serpiginous enhancing vessels at the cervicomedullary junction, indicating a vascular malformation, Figure 2a, b. The nidus measured approximately 24 mm (anteroposterior) × 24 mm (transverse) × 34 mm (craniocaudal). The AVM received arterial supply from multiple vessels including the distal right vertebral artery near its junction with the left vertebral artery (at the formation of the basilar artery) and anterior spinal artery (Figure 2c). Venous drainage was via a prominent vein into bilateral sigmoid sinuses as shown in Figure 2d, with associated venous hypertension demonstrated by contrast reflux into the anterior spinal veins and deep cervical veins.

**Figure 3:**
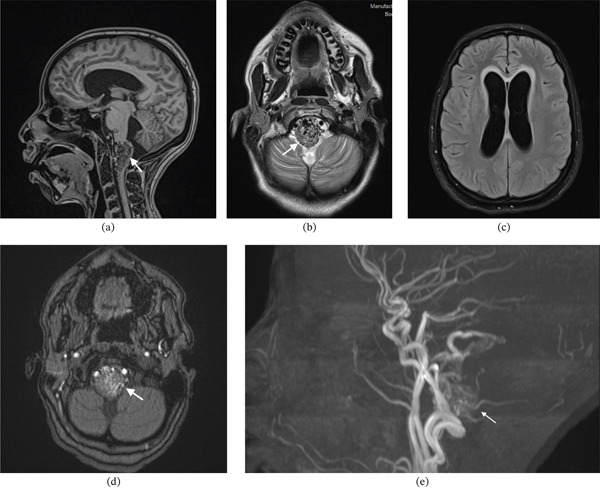
MRI of the brain demonstrating the AVM. (a) Sagittal T1‐weighted image shows multiple serpiginous flow voids at the cervicomedullary junction with focal expansion and anterior displacement of the medulla. (b) Axial T2‐weighted image also shows the abnormal flow voids. (c) Axial FLAIR image demonstrates acute hydrocephalus with ventricular enlargement. (d) Axial postcontrast T1‐weighted image confirms an enhancing vascular nidus. (e) 3D time‐of‐flight MR angiogram delineates the nidus and its arterial feeders.

**Figure 4:**
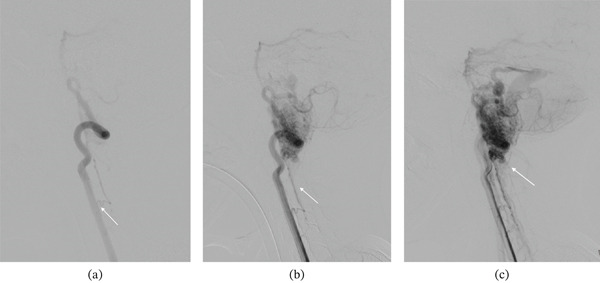
Digital subtraction angiography (lateral projections). (a) Early arterial phase demonstrating a prominent feeder arising from the anterior spinal artery (ASA). (b) Midarterial phase showing the nidus in continuity with the anterior spinal artery on the lateral projection. (c) Progressive opacification of the nidus with increasing filling of the anterior spinal artery on sequential lateral imaging.

**Figure 5:**
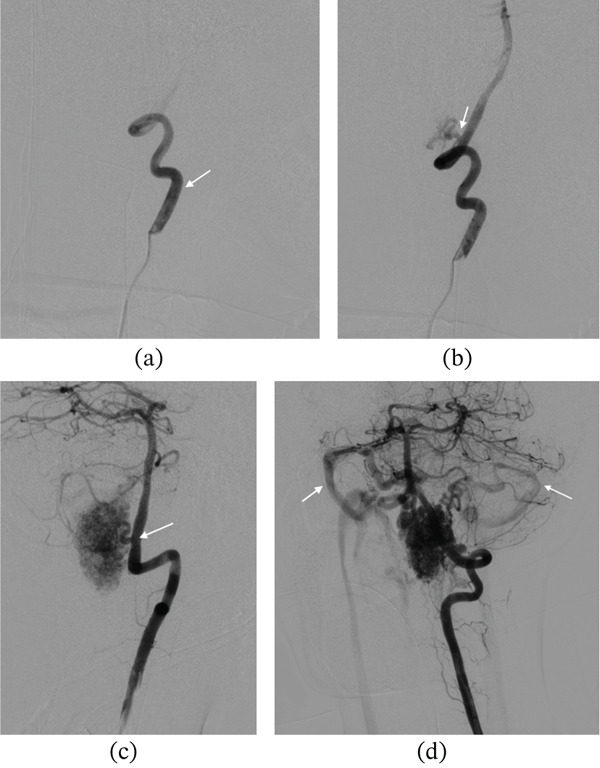
Digital subtraction angiography of a posterior fossa arteriovenous malformation (AVM). (a, b) Anteroposterior left vertebral artery injection further demonstrating arterial feeders to the nidus. (c) The posterior inferior cerebellar artery (PICA) is identified (white arrow). (d) Magnified view shows a posterior fossa nidus with early venous drainage into the right and left transverse and sigmoid sinuses (white arrows).

MRI of the brain provided further detail. Multiple serpiginous flow voids were observed at the cervicomedullary junction, with slight anterior displacement of the medulla, consistent with a vascular malformation (Figure 3a,b). An acute obstructive hydrocephalus was evident on axial FLAIR imaging (dilated ventricles on Figure 3c). Postcontrast T1‐weighted sequences confirmed an enhancing vascular nidus at the cervicomedullary junction (Figure 3d). A 3D time‐of‐flight (TOF) MR angiogram delineated the nidus and its arterial feeders (Figure 3e). Finally, DSA was performed for definitive characterisation, confirming a juvenile Type IIa posterior fossa arteriovenous malformation centred at the cervicomedullary junction, measuring approximately 2.5 × 3.5 cm. Angiography demonstrated a prominent feeder arising from the anterior spinal artery in the early arterial phase, with a nidus in direct continuity and progressive opacification on sequential imaging (Figure 4). Additional posterior fossa angiography delineated arterial supply from the left vertebral artery, including the posterior inferior cerebellar artery, with early venous drainage into the transverse and sigmoid sinuses (Figure 5).

Given these findings, a diagnosis of a juvenile Type IIa AVM at the cervicomedullary junction was confirmed. The lesion was classified as Spetzler–Martin (SM) Grade IV and Lawton–Young supplementary Score 2.

The patient was admitted for close neurologic monitoring and managed with strict blood pressure control and analgesia. A multidisciplinary team involving neurosurgery, interventional neuroradiology and radiation oncology reviewed the case. Surgical resection or endovascular embolisation was deemed prohibitively high‐risk due to the nidus′s location adjacent to the brainstem and its diffuse angioarchitecture. Gamma Knife radiosurgery was considered as a less invasive option, but it was ultimately deferred in light of the lesion′s diffuse nature and eloquent location (within the medulla). The decision was made to pursue conservative management with follow‐up imaging, given that the immediate threat from the haemorrhage had been addressed and any intervention carried significant risk.

## 3. Discussion

Posterior fossa AVMs, though rare, are clinically important because of their location in tight anatomical confines where even small bleeds can be catastrophic. Stapf et al. [4] noted that infratentorial (posterior fossa) AVMs rupture more frequently than supratentorial lesions, and Gross and Du [5] found annual haemorrhage rates as high as 6% in these cases. This propensity for haemorrhage explains why our patient presented with a subarachnoid bleed despite her young age and otherwise good health.

Juvenile AVMs are particularly complex lesions. Lasjaunias et al. [3] described Type IIa juvenile AVMs as large, diffuse lesions fed by multiple arterial pedicles with complicated venous drainage. These features make curative treatment exceptionally difficult. In our case, the cervicomedullary location and diffuse nidus were characteristic of this subtype and informed the decision to avoid any immediate intervention.

Imaging was central to the diagnosis and management plan. The initial CT confirmed the presence of haemorrhage, whereas CTA suggested abnormal serpiginous vessels at the brainstem–upper cervical region. MRI provided crucial anatomical detail, defining the relationship of the nidus to the medulla and helping to exclude alternative diagnoses such as a cavernous malformation. DSA remains the gold standard for characterising AVMs because it delineates dynamic flow patterns, arterial feeders and venous drainage in detail, which was essential in this case for classification and risk assessment.

Treatment of posterior fossa AVMs is one of the most challenging aspects of their management. Surgery can be curative for AVMs, but brainstem involvement makes surgical intervention extremely high risk; Abla et al. [6] reported that approaches to lesions in the brainstem carry a high likelihood of permanent neurological deficits. Less invasive approaches have been explored. Sun et al. [7] demonstrated that even cerebellar AVMs can be treated via endovascular embolisation in selected cases, though with limited success rates. Pezeshkpour et al. [8] similarly advocated for multimodal strategies in paediatric AVM patients, combining staged embolisation with subsequent radiosurgery in carefully chosen cases. However, it must be emphasised that embolisation is often incomplete, and Sato et al. [9] have detailed the significant complication risks of endovascular treatment, especially when deep perforating arteries are involved. Radiosurgery has emerged as an option for AVMs located in deep or eloquent regions (such as the brainstem) where surgery is too dangerous. The International Stereotactic Radiosurgery Society′s recent guideline [10] supports considering stereotactic radiosurgery (SRS) for higher‐grade AVMs (SM Grades III–V) in certain circumstances, although the evidence base remains limited. Notably, Cohen‐Inbar et al. [11] reported that for cerebellar AVMs, radiosurgical obliteration rates can approach 70% when adequate doses (≥ 18 Gy) are delivered. Nevertheless, radiosurgery is not without risk, as patients remain at risk of haemorrhage during the latency period before obliteration, and they may experience radiation‐induced neurotoxic effects, particularly in critical locations like the brainstem.

SRS was carefully considered in this case but ultimately deferred following multidisciplinary discussion. The diffuse nidus of the arteriovenous malformation and its eloquent cervicomedullary location were key limiting factors, as even targeted radiation would pose a substantial risk to adjacent critical brainstem structures, including the medulla oblongata, with potential for severe neurological deficit. Single‐session SRS was not favoured given the lesion′s extent, diffuse juvenile angioarchitecture and associated high‐flow shunting, all of which reduce the likelihood of safe and effective obliteration while increasing the risk of radiation‐induced toxicity [12, 13]. Fractionated or staged SRS approaches were discussed conceptually; however, the anticipated benefit remained limited in the context of the lesion configuration and proximity to eloquent neural tissue, with a persistent risk of haemorrhage during the latency period [13].

Preradio surgical embolisation was also considered as a potential adjunct to reduce flow; however, the arterial supply from the anterior spinal axis and the associated risk of catastrophic ischaemic injury rendered this approach unfavourable [12]. Microsurgical resection was similarly deemed prohibitively high risk due to the deep‐seated cervicomedullary location and the likelihood of severe morbidity, including quadriplegia or death.

To further contextualise management decisions, established grading systems were applied. Based on the angiographic findings, the lesion was assigned a SM Grade IV, reflecting a nidus size of approximately 2.5–3.5 cm (Score 2), eloquent location within the cervicomedullary junction (Score 1) and deep venous drainage via perimedullary and dural venous systems (Score 1). This high SM grade is associated with a substantially increased risk of morbidity with microsurgical intervention. In addition, the Lawton–Young supplementary grading system was applied, incorporating patient age (< 20 years; Score 1), haemorrhagic presentation (Score 0) and diffuse (noncompact) nidus morphology (Score 1), yielding a supplementary score of 2. The combined grading profile (SM IV, Supplementary 2) further supports the high‐risk nature of this lesion and reinforces the unfavourable risk benefit ratio for intervention [14].

Taking these factors into account, the balance of potential benefit versus harm did not favour intervention at this stage. Conservative management was therefore selected despite the haemorrhagic presentation, reflecting a careful consideration of the natural history risk of rebleeding versus the substantial risks associated with intervention [15]. The patient was counselled extensively regarding the risks and alternatives and ultimately declined SRS. This case underscores the importance of individualised, multidisciplinary decision‐making in the management of complex, high‐risk vascular lesions.

Accordingly, conservative management in this patient was coupled with active surveillance rather than passive observation. Planned follow‐up consisted of ongoing neurological review and contrast enhanced MRI/MRA at 6 months, followed by annual imaging if stable. Surveillance was intended to detect interval haemorrhage, nidus enlargement, worsening venous congestion or the development of new high‐risk angioarchitectural features. Contemporary consensus recommendations emphasise the role of MRI/MRA in longitudinal surveillance, with advanced techniques such as 4D MRA and arterial spin labelling improving haemodynamic assessment, whereas DSA remains the reference standard for definitive angioarchitectural characterisation and is reserved for cases where treatment decisions are being reconsidered or imaging changes are detected [12, 16, 17].

Rediscussion at multidisciplinary review would be triggered by rebleeding, interval enlargement of the nidus, progressive venous hypertension or congestion, the development of a flow‐related or intranidal aneurysm, or worsening neurological symptoms. In such circumstances, repeat angiographic evaluation and reconsideration of intervention would be warranted, particularly if evolving endovascular, radiosurgical or microsurgical techniques altered the risk–benefit profile. Given the patient′s young age and the cumulative lifetime risk of haemorrhage associated with arteriovenous malformations, long‐term surveillance was considered essential even in the absence of immediate intervention [12, 15].

Cervicomedullary junction AVMs represent a rare and complex subset of intracranial vascular malformations. Despite the inherent risk of haemorrhage, intervention may carry even greater morbidity due to proximity to critical brainstem structures. This case highlights the importance of multimodality imaging, structured risk stratification and multidisciplinary decision‐making in guiding patient‐centred management.

## 4. Conclusion

Cervicomedullary junction arteriovenous malformations represent a rare and complex subset of intracranial vascular lesions, posing a significant therapeutic dilemma. Although the risk of haemorrhage is substantial, intervention may confer equal or greater morbidity due to the lesion′s proximity to critical brainstem structures and its often diffuse angioarchitecture. This case underscores the essential role of multimodality imaging in accurate diagnosis and risk stratification, as well as the importance of multidisciplinary evaluation in guiding management. In selected high‐risk cases, conservative management with structured surveillance may represent the most appropriate strategy. Ultimately, such cases highlight the need for continued refinement of evidence‐based approaches to optimise outcomes in this challenging patient population.

## Author Contributions

A. Rumhumha—concept and initial drafting of manuscript. R. Minty—imaging interpretation, drafting manuscript and figure preparation. A. Lupuwana—imaging interpretation and figure preparation. A. Ranchod—critical revision of the manuscript. W. Nkosi—critical revision of the manuscript.

## Funding

No funding was received for this manuscript.

## Disclosure

M. Ramantsi provided final approval of the manuscript.

## Consent

Written informed consent was obtained from the patient for publication of this case and the accompanying images.

## Conflicts of Interest

The authors declare no conflicts of interest.

## Data Availability

The data that support the findings of this study are available on request from the corresponding author. The data are not publicly available due to privacy or ethical restrictions.

## References

[1] Spetzler R. F. and Martin N. A. , A Proposed Grading System for Arteriovenous Malformations, Journal of Neurosurgery. (1986) 65, no. 4, 476–483, 10.3171/jns.1986.65.4.0476, 2-s2.0-0022465686. 3760956

[2] Arnaout O. M. , Gross B. A. , Eddleman C. S. , Bendok B. R. , Getch C. C. , and Batjer H. H. , Posterior Fossa Arteriovenous Malformations, Neurosurgical Focus. (2009) 26, no. 5, 10.3171/2009.2.FOCUS0914, 2-s2.0-70349740757. 19408990

[3] Lasjaunias P. , Manelfe C. , and Chiu M. , Le livre du qaras*ţ* *u*n de Tābit ibn Qurra, Archive for History of Exact Sciences. (1974) 13, no. 4, 325–347, 10.1007/BF00327299, 2-s2.0-34250424102.

[4] Stapf C. , Mohr J. P. , Pile-Spellman J. , Solomon R. A. , Sacco R. L. , and Connolly E. S. , Epidemiology and Natural History of Arteriovenous Malformations, Neurosurgical Focus. (2001) 11, no. 5, 10.3171/foc.2001.11.5.2. 16466233

[5] Gross B. A. and Du R. , Natural History of Cerebral Arteriovenous Malformations: A Meta-Analysis, Journal of Neurosurgery. (2013) 118, no. 2, 437–443, 10.3171/2012.10.JNS121280, 2-s2.0-84873695892, 23198804. 23198804

[6] Abla A. A. , Turner J. D. , Mitha A. P. , Lekovic G. , and Spetzler R. F. , Surgical Approaches to Brainstem Cavernous Malformations, Neurosurgical Focus. (2010) 29, no. 3, 10.3171/2010.6.FOCUS10128, 2-s2.0-77956209265. 20809766

[7] Sun Y. , Chang Q. , You W. , Liu P. , Lv X. , Li Y. , and Lv M. , Endovascular Treatment of Cerebellar Arteriovenous Malformations: A Single-Center Experience of 75 Consecutive Patients, Neurology India. (2020) 68, no. 2, 440–447, 10.4103/0028-3886.284347, 32415021. 32415021

[8] Pezeshkpour P. , Muthusami P. , Dmytriw A. A. , Shroff M. M. , Phan P. , Dirks P. , Kulkarni A. V. , and Muthusami P. , Treatment Strategies and Related Outcomes for Brain Arteriovenous Malformations in Children: A Systematic Review and Meta-Analysis, American Journal of Roentgenology. (2020) 215, no. 2, 472–487, 10.2214/AJR.19.22443. 32507016

[9] Sato K. , Terada T. , and Endo S. , Complications of Endovascular Treatment for Brain Arteriovenous Malformations: An Updated Review, American Journal of Neuroradiology. (2020) 41, no. 5, 833–839, 10.3174/ajnr.A6470. PMC7144660 32193193

[10] Graffeo C. S. , Kotecha R. , Sahgal A. , Fariselli L. , Gorgulho A. , Levivier M. , Ma L. , Paddick I. , Regis J. , Sheehan J. P. , Suh J. H. , Yomo S. , and Pollock B. E. , Stereotactic Radiosurgery for Intermediate (III) or High (IV–V) Spetzler–Martin Grade Arteriovenous Malformations: International Stereotactic Radiosurgery Society Practice Guideline, Neurosurgery. (2025) 96, no. 2, 298–307, 10.1227/neu.0000000000003102, 38989995. 38989995

[11] Cohen-Inbar O. , Starke R. M. , Kano H. , Bowden G. , Huang P. , Rodriguez-Mercado R. , Almodovar L. , Grills I. S. , Mathieu D. , Silva D. , Abbassy M. , Missios S. , Lee J. Y. K. , Barnett G. H. , Kondziolka D. , Lunsford L. D. , and Sheehan J. P. , Stereotactic Radiosurgery for Cerebellar Arteriovenous Malformations: An International Multicenter Study, Journal of Neurosurgery. (2017) 127, no. 3, 512–521, 10.3171/2016.7.JNS161208, 2-s2.0-85028772691, 27689461. 27689461

[12] Samaniego E. A. , Dabus G. , Meyers P. M. , Kan P. T. , Frösen J. , Lanzino G. , Welch B. G. , Volovici V. , Gonzalez F. , Fifi J. , Charbel F. T. , Hoh B. L. , Khalessi A. , Marks M. P. , Berenstein A. , Pereira V. M. , Bain M. , Colby G. P. , Narayanan S. , Tateshima S. , Siddiqui A. H. , Wakhloo A. K. , Arthur A. S. , Lawton M. T. , and ARISE I Consortium , Most Promising Approaches to Improve Brain AVM Management: ARISE I Consensus Recommendations, Stroke. (2024) 55, no. 5, 1449–1463, 10.1161/STROKEAHA.124.046725, 38648282. 38648282

[13] Starke R. M. , Kano H. , Ding D. , Lee J. Y. K. , Mathieu D. , Whitesell J. , Pierce J. T. , Huang P. P. , Kondziolka D. , Yen C. P. , Feliciano C. , Rodgriguez-Mercado R. , Almodovar L. , Pieper D. R. , Grills I. S. , Silva D. , Abbassy M. , Missios S. , Barnett G. H. , Lunsford L. D. , and Sheehan J. P. , Stereotactic Radiosurgery for cerebral Arteriovenous Malformations: evaluation of Long-Term Outcomes in a multicenter cohort, Journal of Neurosurgery. (2017) 126, no. 1, 36–44, 10.3171/2015.9.JNS151311, 2-s2.0-85019455194, 26943847. 26943847

[14] Lawton M. T. , Kim H. , McCulloch C. E. , Mikhak B. , and Young W. L. , A Supplementary Grading Scale for Selecting Patients With Brain Arteriovenous Malformations for Surgery, Neurosurgery. (2010) 66, no. 4, 702–713, 10.1227/01.NEU.0000367555.16733.E1, 2-s2.0-77949943392, 20190666. 20190666 PMC2847513

[15] Derdeyn C. P. , Zipfel G. J. , Albuquerque F. C. , Cooke D. L. , Feldmann E. , Sheehan J. P. , Torner J. C. , and American Heart Association Stroke Council , Management of Brain Arteriovenous Malformations: A Scientific Statement for Healthcare Professionals From the American Heart Association/American Stroke Association, Stroke. (2017) 48, no. 8, e200–e224, 10.1161/STR.0000000000000134, 2-s2.0-85021261552, 28642352. 28642352

[16] De Simone M. , Fontanella M. M. , Choucha A. , Schaller K. , Machi P. , Lanzino G. , Bijlenga P. , Kurz F. T. , Lövblad K. O. , and De Maria L. , Current and Future Applications of Arterial Spin Labeling MRI in Cerebral Arteriovenous Malformations, Biomedicines. (2024) 12, no. 4, 10.3390/biomedicines12040753. PMC11048131 38672109

[17] Kolahi S. , Tahamtan M. , Sarvari M. , Zarei D. , Afsharzadeh M. , Firouznia K. , and Yousem D. M. , Diagnostic Performance of TOF, 4D MRA, Arterial Spin-Labeling, and Susceptibility-Weighted Angiography Sequences in the Post-Radiosurgery Monitoring of Brain AVMs, American Journal of Neuroradiology. (2025) 46, no. 1, 57–65, 10.3174/ajnr.A8420, 39025641. 39025641 PMC11735419

